# Dynamic Magnetic Responsive Wall Array with Droplet Shedding-off Properties

**DOI:** 10.1038/srep11209

**Published:** 2015-06-10

**Authors:** Lei Wang, Miaoxin Zhang, Weiwei Shi, Yongping Hou, Chengcheng Liu, Shile Feng, Zhenyu Guo, Yongmei Zheng

**Affiliations:** 1 Key Laboratory of Bio-Inspired Smart Interfacial Science and Technology of Ministry of Education, School of Chemistry and Environment, Beihang University, Beijing, 100191 (P. R. China)

## Abstract

**Directional control of droplets on a surface is an important issue for tasks of long-range liquid-transport, self-cleaning and water repellency. However, it is still challenging to control the structure motions in orientations so as to control the shedding-off of droplets. Herein, we report a novel dynamic magnetic responsive wall (DMRW) array on PDMS (polydimethylsiloxane) -based surface. The walls can easily tilt through the effect of the external magnet because of the magnetic material in the DMRW. The droplets can be shed off directionally on the surface. Particularly, with the shape recovery and flexible properties, it achieves simultaneous control of the tilt angles (0-60°) of DMRW for shedding-off of droplets with different volumes (1-15** **μL) under magnetic action on DMRW. The mechanism of droplet shedding-off on DMRW is elucidated by theory of interfaces. It offers an insight into design of dynamic interface for water repellency. This strategy realizes the preparation of multifunctional, tunable and directional drive functions.**

Directional control of droplets on patterned surfaces is an important issue for tasks of long-range liquid-transport, self-cleaning and water repellency^1^,^2^,^3^. On as-fabricated surfaces, droplets motion may be effectively controlled by chemical gradient resulting from introduction of two scientific models, i.e., controllable liquid motion and self-propelled motion^4^,^5^,^6^,^7^,^8^. Natural biological materials, e.g., lotus leaf, Indian cress leaf, lady’s mantle leaf, gecko, butterfly wing, etc.^3^,^9^,^10^,^11^,^12^,^13^,^14^,^15^, display an excellent controlling to wettability of surface, which offer significant theories to understand the nature and help design materials. In general, droplets on superhydrophobic symmetric or anisotropic patterned surfaces take on different shapes, which are determined by minimal energy principle^16^. However, on fixed-structured surface, the droplets always tend to reach the steady state, and under traditional external force, it is hard to overcome the energy barriers introduced by channel or grooves features. Up to now, it is still challenging to control the structure motions in orientations so as to control the directional shedding-off of droplets on a surface. Herein, we report a novel dynamic magnetic controlled flexible-wall (DMRW) on PDMS (polydimethylsiloxane) -based surface. The walls can easily tilt through the effect of the external magnet because of the magnetic material in the DMRW. The droplets can be shed off directionally on the surface. Particularly, with the shape memory and flexible properties, it achieves simultaneous control of the tilt angles of DMRW and its ability to drive the droplets with different volumes. This strategy realizes the preparation of multifunctional, tunable and directional drive functions.

In the experiment, the DMRWs are fabricated by integrating the methods of soft-replica and crystal growth (see Fig. 1a, the schematic of fabrication process)^16^,^17^. Vertical flexible walls are fabricated with width of 200 μm, heights of 500 μm, and spacing of 200 μm. To lower the retention force of liquid on the surface, we modified the surface with Heptadeca Fluorodecyltri-propoxysilane (FAS-17) by physical vapor deposition. Figure 1b,c are low magnification of top and side view of the structure. High magnification view of the surface is recorded (Fig. 1d), as the surface is covered by ZnO nano-hairs. ZnO nano-structures enhance the roughness of DMRW and provide much pocketed air between droplet and DMRW, which result in high contact angle and low adhesion^17^. To investigate the component and distribution of elements on DMRW, we analyze the cross section of it with energy dispersive spectroscopy (EDS). The EDS results are consistent with the composition of DMRW, i.e., PDMS (Si, O, C), ZnO nano-hairs (Zn, O), Fe (Fe) (Fig. 1e). The distribution of chemical elements, such as Fe, Zn, C and Si are observed by EDS (Fig. 1e, and also see Fig. S1).

The motion of DMRWs is induced via an externally applied magnetic field generated by neodymium-iron-boride permanent magnet^18^,^19^. In our experiment, the magnetism acts on Fe micro-particles in the DMRWs, and makes the DMRWs tilt into angles. As known, Iron (Fe) as paramagnetic material is used for structure-driven. With the increase of external magnetic field strength (magnet moved from infinity in towards the DMRWs), Fe will be magnetized and attracted by the neodymium iron boron magnet. Fe shows magnetism when external magnet gets close to it. The structures will tilt and the tilt angle depends on the intensity of magnetic field. When the external magnetic field decrease or vanished, the structure will recover to the initial state due to the elastic property of PDMS. The tilt degree of the DMRWs is recorded by a high-speed CCD camera (Phantom v 9.1, America). We can observe the tilt angles (*β*_tilt_ ) of DMRWs versus the magnetic intensity (*I*_m_) as shown in Fig. 2. Figure 2a show the schematic of driven motion. (As distance between magnet and substrate decreases, the strength of magnetic fields increase and the tilt angle of DMRWs becomes larger, see Frame 2a1–a3). Figure 2b–d shows the optical images from the top view (the left) and the side view (the right) on the tilt degree of DMRW. For observations of magnetic-driven, two directions are defined: direction 1 represents the direction towards the tilt direction; direction 2 indicates the direction against direction 1. Figure 2e shows the sequent photos of DMRWs with tilt angles, *β*_tilt_, ranging from 0° to 60° from side view. The relationship diagram of magnetic strength to tilt angles of DMRWs is recorded, and the tilt angles increase with the gradually increase of magnetic strength (Fig. 2e). The magnetic strength ranging from 0 to 1 Tesla, generates different tilt angle on DMRWs, e.g., when the strength reach to 0.5 Tesla, the tilt angle increases to 30°. With the tilt effect of DMRWs, the anisotropic strategy trend is more distinct. The tilt angle (βtilt) of DMRW increases with the magnetic field strength (Im) based on the measurements. (Figure S3). This investigation realizes the dynamic structure array surface controlled by magnetic action, where magnetic strength can be in direct proportion to tilt angle of DMRWs.

To investigate the impact of dynamic structure to the droplet, a droplet with volume of 3 μL is applied for test. With the increase of tilt angle of micro-structure, the droplet rolls off the surface with positive direction synchronically (Frame 1–3 in Fig. 3a,b). The motion of droplet would be controlled in opposite directions (Frame 4 in Fig. 3a,b). Figure 3c further illustrates the relationship between the altering volumes of droplets and tilt angles of wall array. The insets are the optical images of droplet shedding-off at critical tilt angles of wall array for given volumes of droplets. From the curve, we find that the critical tilt angle of DMRWs is not linear with the volume of droplet, which may be caused by three factors: 1. With increasing volume of droplet (the volume range from 3 μL to 15 μL), the contact area increases, inducing larger retention force to opposite the driven motion; 2. Small droplet with volume ranging from 1 μL to 3 μL on DMRWs will pin between two or three DMRWs and add the difficulty for driven; 3. Smaller droplet with the volume less than 1 μL on the DMRWs will pin between two DMRWs and fail in driven. The critical tilt angles for driving droplets change from 17° to 55° and the limitation of driven volume ranges from 1 μL to 15 μL.

To further study the droplet motion on variable structure, we observe the cross section of driven process and intrinsic contact angle and advancing/ receding contact angles on variable DMRWs by optical contact angle meter system and a droplet (deionized water) with volume of 3 μL is deposited on the surface for driven test (Fig. 4a). The motion of droplet is found to be well-correlated with the behavior of DMRW (see Supplementary Movie S1). We use the magnet to investigate the effect of micro-structure tilt angle, *β*_tilt_, the advancing/ receding angles, θ_A_ and θ_R_, on variable DMRWs. At initial time, without magnetic effect, the droplet shape is symmetric on four upright walls (Fig. 4a,f). The intrinsic CA of droplet on DMRWs is 152° and the sliding-off angle (SA) is 2.3° (Fig. S4a and 3b)^7^. The DMRW without ZnO nano-hairs fails in large CA and small SA (Fig. S4c and 3d). The three-phase contact lines (TCLs, the solid black lines) are illustrated in Fig. 4f–h (also see Fig. S5). On the symmetric surface, the TCLs on each side are isotropic (Fig. 4f, the solid black lines on DMRW-3, -4, -5 and -6). With increasing magnetic intensity (*I*_m_), the DMRW tilt angle (*β*_tilt_) and the discrepancy Δ(θ_A_−θ_R_) between advancing contact angle (ACA, right CA) and receding contact angle (RCA, left CA) of droplet increases^17^, i.e., there is *I*_m_ ∝ *β*_tilt _∝ Δ(θ_A_−θ_R_) (see Figure S3). The TCLs on DMRWs change as follows: the TCL on DMRW-6 in direction 1 (tilt direction of DMRW is defined as direction 1) (Fig. 4g, the solid black lines on DMRW-5 and -6) increases, but in direction 2, the TCL on DMRW-3 decreases (Fig. 4g, the solid black lines on DMRW-3). The retention force ranges from ~2 μN to ~18 μN and friction coefficient (u) ranges from ~0.05 to ~0.6 for the tilted angles of DMRWs from 0 to 50° (Figure S6 and S7). In direction 1, the retention force decreases in the growth of tilt angle. In direction 2, the retention force increases in the growth of tilt angle. As a result, the diversity of TCL on the both sides of droplet center is formed and it makes the droplet in a metastable state. In our observation, the retention forces of liquid/solid on DMRW-3 and DMRW-6 act on the droplet can be cooperated with each other. The tilt effect induces the increase of TCL on DMRW-6 which raises an advancing force to driven droplet (i.e., tilted DMRW drive the droplet toward direction 1)^20^. In the mean time, the TCL on DMRW-3 decreases to aid the movement of droplet. When the advancing force of DMRW-6 becomes larger than the receding force of DMRW-3, i.e., the advancing contact angle is larger than the receding contact angle. The droplet sheds off from DMRW-3 to DMRW-6. It is estimated that when the DMRWs tilt angle increases to 17°, the discrepancy of ACA and RCA become more than 7° (Fig. 4c,g). When the DMRWs reach to a critical angle, the barycenter moves along direction 1 and the TCL on DMRW-3 shrinks rapidly with the move effect. The deformation of droplet becomes maximum, which leads to least stability. In this state, with any disturbance induced by external force, the droplet will break away from DMRW-3 and move along direction 1. The increasing tilt effect generates small disturbances and drives the droplet off at last (Fig. 4d,e,h). The coefficient of rolling friction can be estimated roughly to be ~3.88 × 10^−3^(see Supplementary analysis).

As known, the droplet rolling off, generally induced as a driving force (e.g., gravity^21^), overcomes the retention force of surface resulting from contact angle hysteresis (determined by difference between advancing and receding angle). Here, the driving force is a force of deformation resulting from dynamic structure with tilt degree timely by magnetic action. The dynamic structure causes the deformation of droplet with difference between advancing and receding angle of droplet on the surface. The force for driving a droplet is basically described as follow^22^:
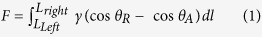
Where *F* is the force for driving a droplet in directional 1; *dl* is the integrating variable along the length from the left to the right of droplet; *γ* is the free energy of liquid at liquid-air interface; θ_*R*_ and θ_*A*_ corresponds to receding angle and advancing angle, respectively. When θ_*R*_ < θ_*A*_, *F* points to direction 1 and increases as the increase of disparity between θ_*R*_ and θ_*A*_. The *F* can be related to tilted angles of DMRWs that are resulted from interaction between Fe particle in DMRW and the intensity of magnetic field (*I*_m_) (Fig. 2e and Figure S3). As we have mentioned above, the retention force in direction 2 decreases as the increase of tilt angle. When *F* is larger than the retention force the droplet overcomes the energy barrier and rolls off directionally. In addition, we also indicate that the DMRWs are covered by ZnO nano-hairs, and modified by low surface energy material. On the surface, the rolling friction is much less than the retention force^23^. When the energy release happens (i.e., the droplet overcomes the adhesion force of DMRW-3), the droplet will roll off directionally on superhydrophobic DMRWs due to the dynamic structure fluctuation under magnetic action. The force (*F*) for driving of droplet can be estimated by θ_*R*_/θ_*A*_, as shown in Table S1. As for driving the droplet (3 μL), *F* ≈ 2.70 μN (see Figure S8).

This work demonstrates the droplet-driven property on dynamic magnetic responsive wall (DMRW) array by soft-replica and enhanced crystal growth methods. Fe micro-particles in DMRWs can be effectively attracted by the magnet and result in tilting the DMRWs. ZnO nano-hairs enhance the roughness of DMRW and FAS-17 lowers the surface adhesion force, which makes the droplet easily roll off the DMRWs with small tilt angle. The droplet with volume of 1 μL–15 μL is easily to be driven off by the tilting DMRW. This investigation offers an exciting insight into the droplet-driven on superhydrophobic and structure-controlled surfaces, and should be useful to design multiple functional surfaces that can achieve directional droplet-driven, liquid long transportation, de-icing, self-recovery, etc.

## Additional Information

**How to cite this article**: Wang, L. *et al.* Dynamic Magnetic Responsive Wall Array with Droplet Shedding-off Properties. *Sci. Rep.* **5**, 11209; doi: 10.1038/srep11209 (2015).

## Supplementary Material

Supplementary Information

Supplementary Information

## Figures and Tables

**Figure 1:**
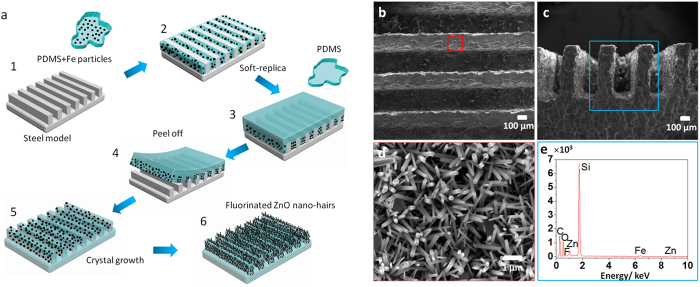
The schematic of fabrication process and the structure of DMRWs. **a**, The illustration of fabrication. The micro-walls of DMRW are fabricated by PDMS with Fe particles and the basement of DMRW is formed by pure PDMS (both with curing agent). After that, the DMRW is modified with ZnO nano-hairs by enhanced crystal growth method. The surface gets superhydrophobic property after being covered by FAS-17. **b-c**, SEM images with low magnification in the top view (b) and side view (c) of the DMRWs. Vertical flexible walls are fabricated with width of 200 μm, heights of 500 μm, and spacing of 200 μm. **d**, SEM image with the magnified view of DMRWs, it is covered by ZnO nano-hairs. The diameters of ZnO range from 50 nm to 80 nm. **e**, the EDS analysis of DMRWs, the mainly chemical constituents are C, O, F, Zn, Fe.

**Figure 2:**
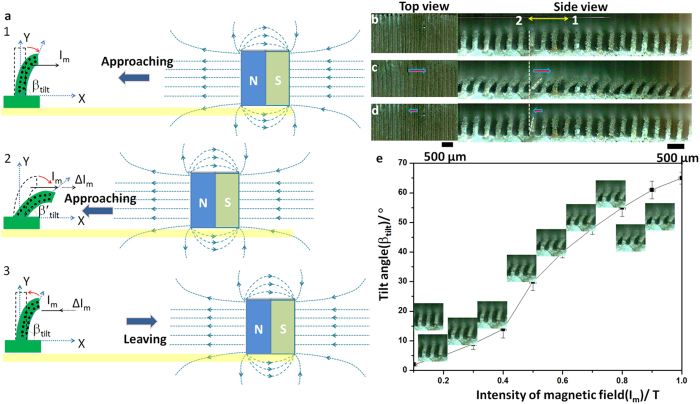
The illustration of the neodymium iron boron magnet used to tilt the DMRWs. **a**, The schematic of the magnetic effect on the tilt angle of DMRW. With the increasing magnetic field by getting closer magnet, the tilt angles β_tilt_ of DMRWs increase. I_m_ and ΔI_m_ are intensity of magnetic field and the increased intensity of magnetic field, respectively. β_tilt_ is the tilt angle of DMRW, the red arrow indicates the dynamic direction of DMRW. Y, X are the vertical and horizontal coordinates, respectively. **b**, **c** and **d**, The top view (the left) and the side view (the right) of the wall array tilt angle process. For observations of magnetic-driven, two directions are defined: direction 1 represents the direction towards the tilt direction; direction 2 indicates the direction against direction 1. **e**, The relationship between magnetic intensity (I_m_) and tilt angles (β_tilt_) of DMRWs. The insets are the sequent photos of DMRWs with change of β_tilt_ from 0° to 60° with the increase of I_m_ from 0 to 1 Tesla.

**Figure 3:**
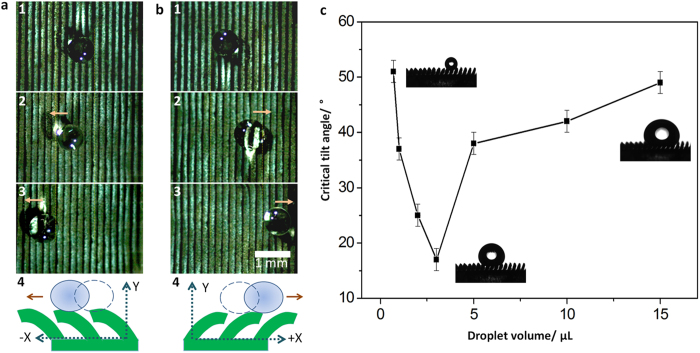
The sequent images of motion of droplet on variable DMRWs. **a**, **b**, Top view of droplet motion along opposite directions on variable DMRWs (Frame 1–3). The schematics of droplet motions are in Frame 4 of a, b, respectively. **c**, The relationship between volumes of droplets and tilt angles of wall array for directional driven of droplets. With the increase volume of droplet (~15 μL > volume > ~ 3 μL), the contact area increase that induces larger retention force to opposite the driven motion; Small droplet (~ 1 μL < volume < ~ 3 μL) on the DMRWs will pin between two or three DMRWs and adds the difficulty for driven. Smaller droplet (volume < ~ 1 μL) will pin between two DMRWs and fails in driven. The critical tilt angles are ranged from 17° to 55°. The limitation of driven volume ranges from 1 μL to 15 μL.

**Figure 4:**
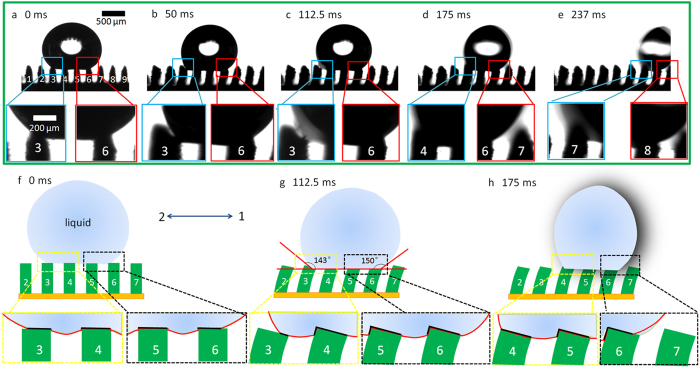
a-e, Optical images of droplet driven on DMRWs. Surface of DMRWs is included 9 micro-walls marked from number 1 to number 9. As the magnet getting closer, the tilt angles increase. The discrepancy between advancing contact angle (ACA) and receding contact angle (RCA) increase induced by the tilt effect and CA hysteresis. The CA discrepancy increases from 0° to 7° (see the inset of **a**, **b**, **c**, **d** and **e**, the left frame is RCA and the right frame is ACA). **f**–**h**, Illustration of (**a**), (**c**) and (**e**). The solid red line represents the three-phase contact line, the dotted yellow and black line frame represent the receding and advancing contact area, respectively.
