# Supplementary material for: Dynamic Magnetic Responsive Wall Array with Droplet Shedding-off Properties
Source: Sci Rep. 2015 Jun 10;5:11209. doi: 10.1038/srep11209 (PMC4462108; doi:10.1038/srep11209)
Supplement: Supplementary Information [file srep11209-s1.pdf]

Supplementary Information

Dynamic Magnetic Responsive Wall Array with Droplet Shedding-off Properties

Lei Wang, Miaoxin Zhang, Weiwei Shi, Yongping Hou, Chengcheng Liu, Shile Feng, Zhenyu Guo, Yongmei Zheng*

Key Laboratory of Bio-Inspired Smart Interfacial Science and Technology of Ministry of Education, Department of Chemistry and Environment, Beihang University, Beijing, 100191 (P. R. China)
***e-mail: zhengym@buaa.edu.cn

Supplementary information is included as follows:

Supplementary Methods

Supplementary Figure Legends

Supplementary Movies

Supplementary methods

Preparation of the hierarchical flexible micro-walls: The micro-structures were prepared by integrating machining method and soft-replica. First, the micro-wall-structured steel model with width of 200 μm, height of 500 μm and space of 200 μm was fabricated by machining and it was used as the duplicating model. And then, polydimethylsioxane (PDMS, SYLGARD 184, Dow Corning, USA) pre-polymer was used for duplicating material. PDMS pre-polymer was mixed with hardener in the proportion of 100:6, and they were moved into a vacuum chamber for removing the air bubbles in the PDMS. Divided the PDMS to two portions, one of them was mixed with Fe micro-particles (with average diameter of 10 μm and purity of 99.99%, purchased from Lanyi company and analyzed by XRD (Figure S2)) in proportion of 50:1. PDMS with Fe micro-particles (Fe-in-PDMS) was poured into the grooved steel model and the surplus PDMS outside the grooved structures was cleared by paper. Afterwards, the other portion of PDMS (pure PDMS) was poured onto the surface with the thickness less than 2 mm. The sample was heated in the oven at 60℃ for 2 h to form solidified PDMS elastomer. The purpose of using two kinds of PDMS for duplicating was to keep the flexible property. Peels off the PDMS from model, the flexible micro-walls with Fe micro-particles were obtained.

Synthesis of ZnO nano-hairs and surface treatment. For driving droplet and getting low surface energy, the roughness of surface must be enhanced. Here, we planted ZnO nano-hairs on Micro-structures by a developed crystal growth method. The crystal seed liquid was prepared as follows: 2 g $Zn(AC)_2 \cdot 2H_2O$ (SCRC, A.R.) and 20 mL ethylene glycol-monomethyl ether (SCRS, A.R.) were mixed and stirred till the solute was dissolved completely. Then, added 0.6 g monoethanolamine (Beijing Chemical Plant, A.R.) into the solution and stirred for 5 min. The growth liquid was prepared as follows: 0.35 g hexamethylenetetramine (Beijing YiLi Fine Chemicals Co., LTD, A.R.) and 100 mL deionized water were mixed and stirred till the solute was dissolved. Next, 0.75 g $Zn(NO_3)_2$ $6H_2O$ (SCRS, A.R.) was added in the solution and stirred for 5 min. Covered the substrate with crystal seed liquid by dip coating, and then moved the substrate into an muffle furnace at 320℃ for 1 min. Put the modified substrate into a Teflon reactor with vertical state, and next dumped the growth liquid into the reactor. The reactor was moved into an oven at 85℃ for 10 h, the nano-structured modified DMRWs were prepared. To get low surface energy, the substrate was modified by heptadecafluorodecyltripropoxysilane (FAS-17, Hangzhou Sage Chemical Co., Ltd, A.R.). Placed the substrate into a vacuum dryer and a droplet of FAS-17 was deposited beside the substrate. The vacuum was placed under reduced pressure at -0.1 MPa and kept at 90℃ for 6 h without light in. Thus the flexible droplet-driven wall (DMRW) was obtained successfully. For the control experiment, the Fe-in PDMS micro-walls without ZnO nanostructure can be modified by FAS-17 in the same conditions.

Structure-driven. The micro-structure of DMRWs was driven by increasing external magnetic field strength. Fe

micro-particles were introduced to mix with PDMS for preparing magnetic drive structures. The neodymium iron boron magnet with 1 Tesla (maximum magnetic field), was used for driving structures. In our experiment, Fe as paramagnetic material was used for structure-driven. With the increase of external magnetic field strength (magnet moved from infinity in towards the DMRWs), Fe would be magnetized and attracted by the neodymium iron boron magnet. Fe would show magnetism when external magnet got close to it, and the structure would recover to the initial state due to the elastic property of PDMS after the external magnetic field decrease or vanished. The magnetic response of DMRW can be repeated because Fe micro-particles will be magnetized again and attracted by the magnet when the structures are driven by external magnet. This process was recorded by CCD camera (Phantom v9.1, Vision Research, America). The width of the sample was ~2 cm, it was a small distance and the difference of field strength on each DMRW was not obvious. All the data were obtained via testing at least 50 times to demonstrate the reproductions.

Characterizations. SEM images were obtained by Environmental Scanning Electron Microscopy (ESEM, Quanta FEG 250, FEI) under the voltage of 5~15 kV. The EDS analysis was tested by Scanning Electron Microscopy (INCA Energy 250, OXFORD INSTRUMENTS).The CAs, SAs and droplet-driven were measured by using dataphysics OCA 20 system. The volume of water droplet was 3 μL. Dynamic process of droplet was recorded by using high-speed CCD camera (Phantom v9.1, Vision Research, America). The retention force was tested by a microelecronic balance system (Dataphysics DCAT21, Germany). A spoon with diameter of 2 mm could hold a 3 μL droplet was hung on the balance hook of a microelectronic balance meter. The droplet could contact with the vertical placed sample from horizontal direction. The adhesion force was recorded by software of the system when the droplet moved relatively on the sample. Crystalline of Fe micro-particles were analyzed by XRD (XRD-6100, Shimadzu, Japan).

Supplementary Figure Legends:

Figure S1:

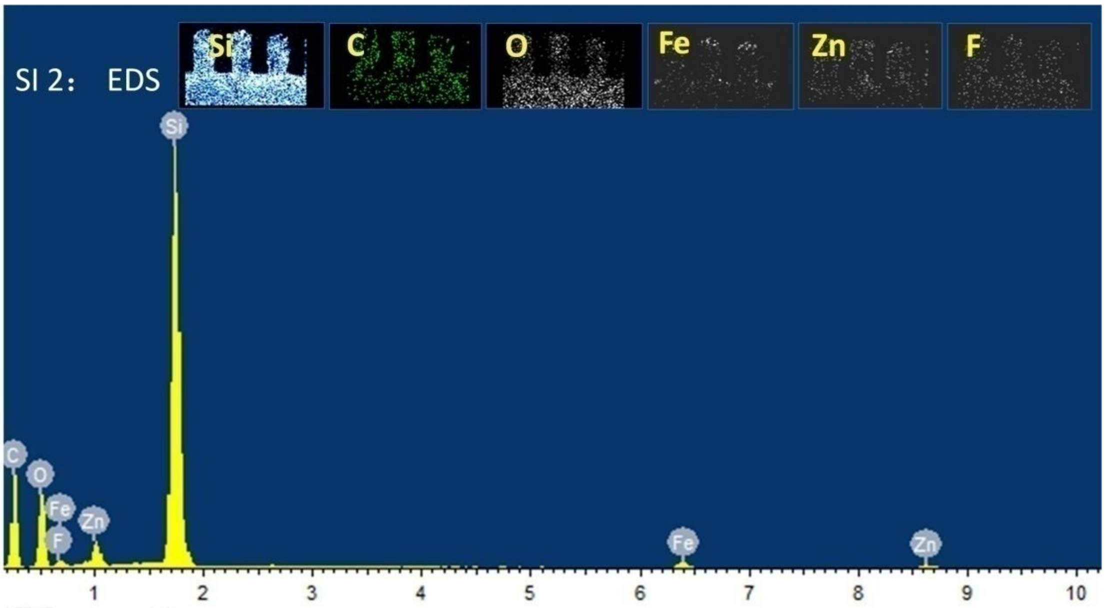

Figure S1. EDS analysis of side view of DMRWs. It showed peaks and distribution (inset images) of elements on DMRWs.

Figure S2:

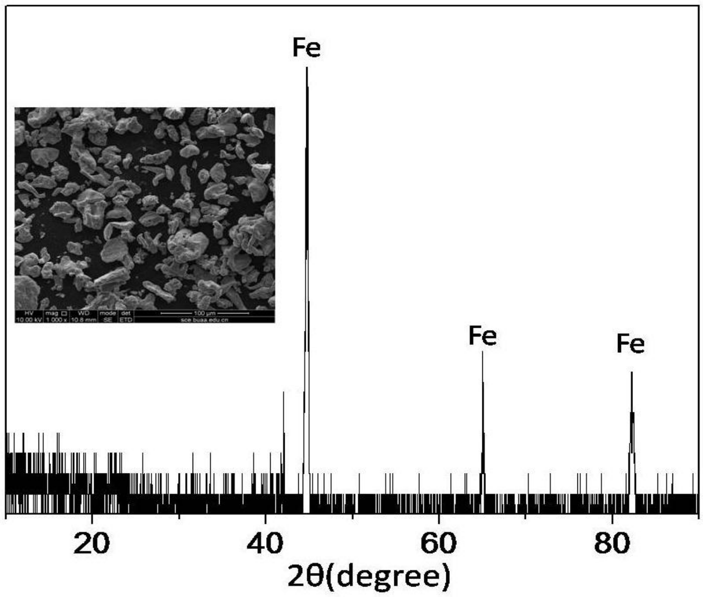

Figure S2. **The X-ray diffraction (XRD) patterns of Fe micro-particles in** DMRW**.** The diffraction peaks can be indentified as pure Fe. The inset is SEM image of Fe particles and the average diameter is ~10 μm.

Figure S3:

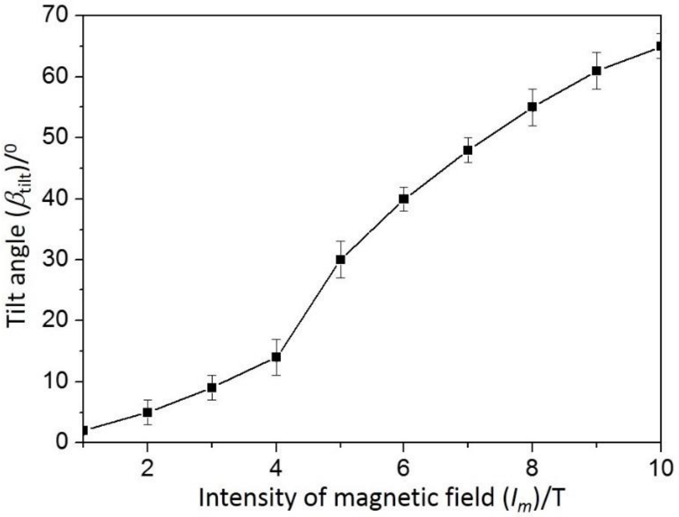

Figure S3. **Relationship magnetic field and tilt angle.** The tilt angle (β_{tilt}) of DMRW increases with the magnetic field strength (I_m) based on the measurements. β_{tilt} is the tilt angle of DMRW; I_m is the intensity of magnetic field (herein, $I_m \geq 0.077$T).

Figure S4:

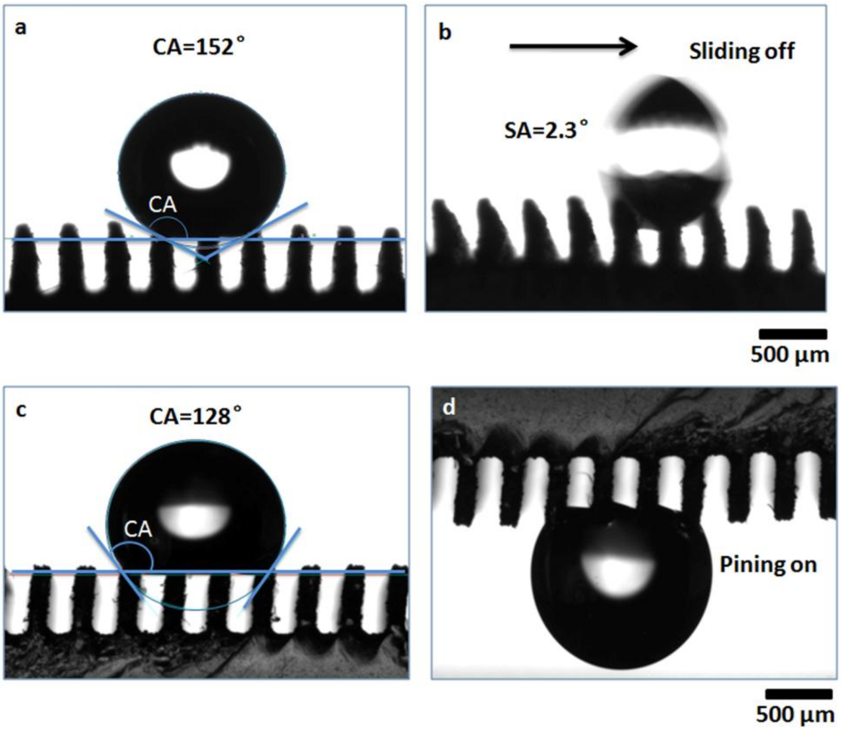

Figure S4. **a-b**, The contact angles and sliding off angles on DMRWs. A droplet with volume of 3 µL has contact angle (CA) of ~152°(a) and sliding off angle (SA) of ~2.3 ° (b) on ZnO and FAS-17 modified DMRWs. **c-d**, The CAs and SCAs on PDMS micro-wall without ZnO nanostructure. The CA of droplet on surface is ~128° (c), and the droplet pins on surface even surface is upside down (d).

Figure S5:

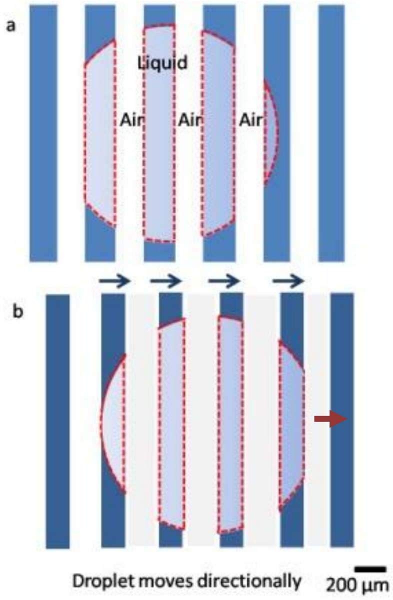

Figure S5. The illustration of the solid/liquid contact area on DMRWs and the red dotted lines represent the three-phase contact lines (TCLs, see broken lines). The blue rectangle respects the DMRWs and the white rectangle respects air between DMRWs, respectively. a) The illustration of droplet on static DMRWs. b) The illustration of the moving droplet on DMRWs under dynamic DMRW (blue arrow indicates the tilt direction of DMRW; red arrow indicates the direction of droplet movement).

Figure S6:

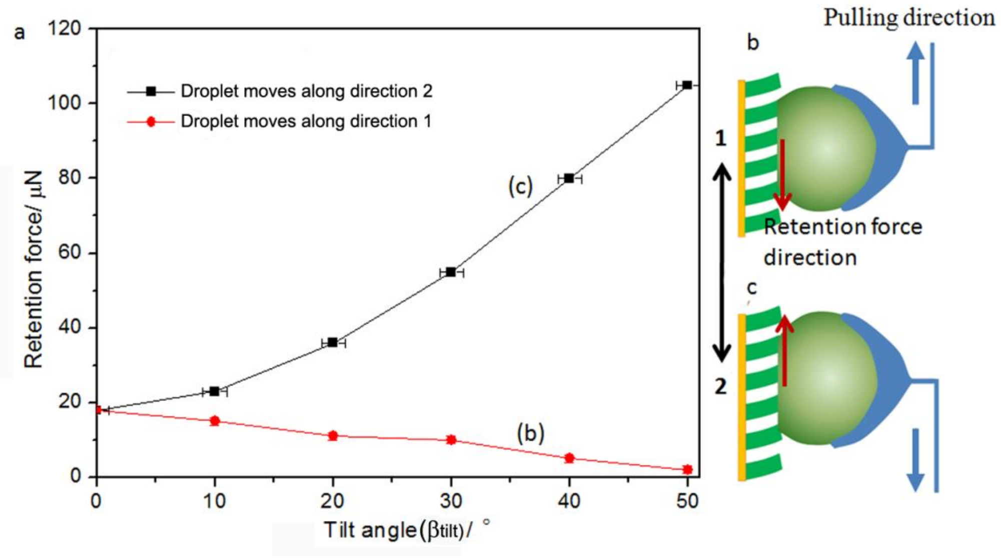

Figure S6. **a**, Curve of relationship between tilt angle of DMRWs and retention force in contrasting directions. As the increase of tilt angles (β_{tilt}), the retention force decreases in direction 1 (b) and increases in direction 2 (c), respectively. The tilt direction of DMRWs is defined as direction 1, and contrasting direction is direction 2 (see two arrows). The volume of droplet is 3 μL. **b**, Illustration of retention force as droplet moves relatively along direction 1. The droplet moves by pulling along direction 1, which is in agreement with tilted direction of DMRWs, the retention force points to the direction 2 against direction 1. **c**, Illustration of retention force as droplet moves relatively along direction 2. The droplet moves by the pulling along direction 2 against the tilted direction of DMRWs, and the retention force points to the direction 2 against direction 1.

Figure S7:

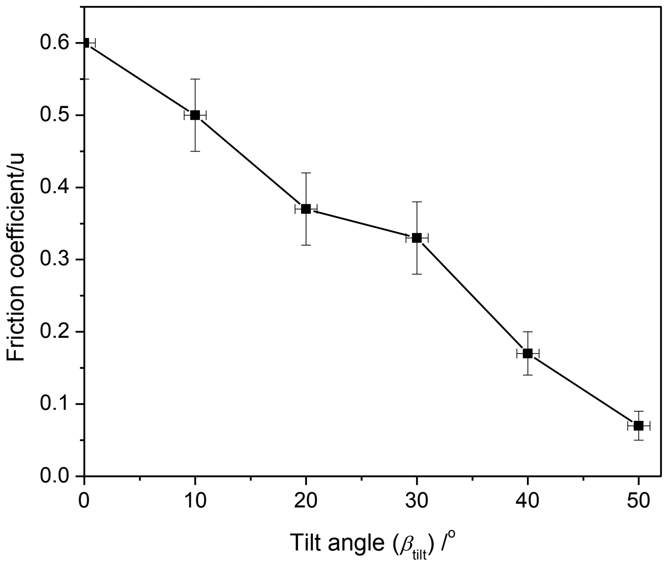

Figure S7. Curve of relationship between tilt angle of DMRWs and friction coefficient (u) in direction 1. As the increase of tilt angles, the friction coefficient decreases in direction 1. The volume of droplet is 3 μL. The friction coefficient (u) can be a ratio of retention force (F_{ret}) to vertical press force (N_{mg}) from gravity, u=F_{ret}/N_{mg}. It is estimated that the u can be ranged in 0.05-0.6 for the tilted angles of DMRWs from 0 to 50° along the direction 1 that droplet sheds off from DMRWs.

Figure S8:

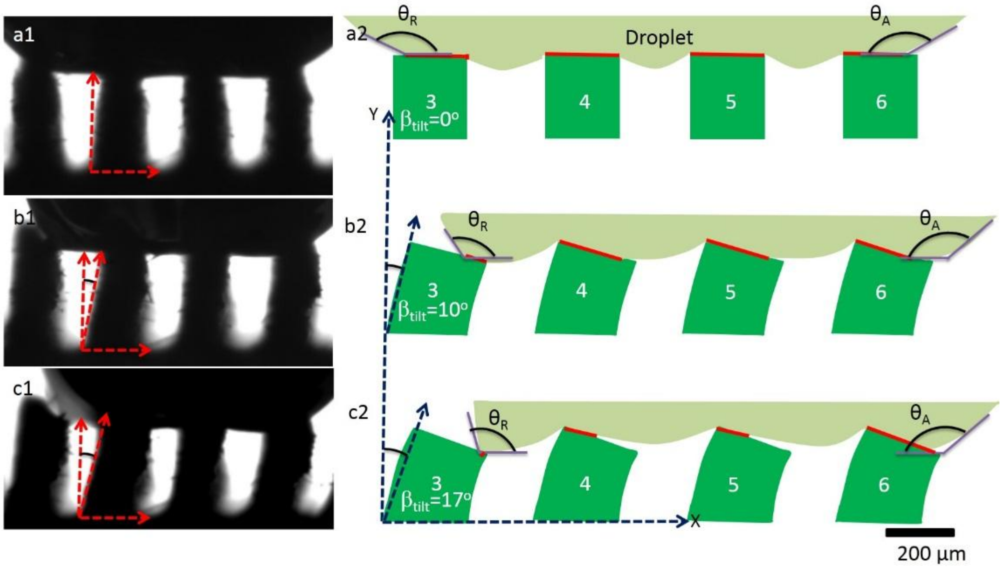

Figure S8. Sequential images of tilting processes and illustration of the change of TCL on the tilt DMRWs. a1, b1 and c1 are the sequential images of tilting processes. a2, b2 and c2 are the illustration of a1, b1 and c1. a is the initial state, b and c are the tilt state with different tilt angles. β_{tilt} is the tilt angle of DMRW, θ_A and θ_R are the advancing and receding contact angle of droplet, respectively. The red line is TCL. The length of TCL (L) decreases with the increase of tilt angle. The length of TCL changes from ~0.7mm to ~0.55 mm with the tilt angle ranging from 0° to 17°. The receding contact decreases with the increase of tilt angle. The scale bar is 200 μm.

Supplementary Analysis on driving force

We can estimate the driving force according to the equation 1: $F = \int_{L_{Left}}^{L_{right}} \gamma(\cos\theta_R - \cos\theta_A)dl$, where F is the force for driving a droplet in directional 1; dl is the integrating variable along the length from the left to the right of droplet; γ is the free energy of liquid at liquid-air interface; θ_R and θ_A corresponds to receding angle and advancing angle, respectively. To estimate the force, the equation 1 can be simplified as $F \approx \gamma L(\cos\theta_R - \cos\theta_A)$, where L is the apparent contact length along the moving direciton (see the illustration of Figure S7). From Figure 4 and Figure S4, contact length and advancing/ receding contact angle (for droplet in volume of 3 μL) can be estimated as shown in Table S1:

Table S1: Analysis of driving force

Tilt angle of DMRW/°	L/mm	θ_A/°	θ_R/°	F/ μN
0	0.70	152	152	0
5	0.63	151	150	0.40
10	0.57	150	148	0.75
15	0.51	150	146	1.37
17	0.55	150	143	2.70

Supplementary analysis on rolling friction coefficient for droplet:

For indicating the coefficient of rolling friction, we first evaluate the acceleration of droplet (with volume of 3 μL). The equation: $V_t^2 - V_0^2 = 2aX$, is introduced. V_t and V_0 are the instantaneous velocity and initial velocity, a is the acceleration, and X is the distance. In the driving movement, $V_t \approx 0.0165$ m/s, $V_t \approx 0.0140$ m/s, X=0.002 m. It is estimated as $a \approx -0.038 \text{m/s}^2$. According to the equation, $f=ma$, in which f is the rolling friction forces, m is the mass of droplet. Thus we can estimate the rolling friction force, $f \approx 1.9\times10^{-7}$ N. Based on the equation of coefficient of rolling friction (η), $\eta = f/mg$. We get coefficient of rolling friction, $\eta = 3.88\times10^{-3}$.

Supplementary Movie:

Movie S1: The droplet shedding off from DMRWs.
