# Supplementary figures and images for: Dynamic Magnetic Responsive Wall Array with Droplet Shedding-off Properties
Source: Sci Rep. 2015 Jun 10;5:11209. doi: 10.1038/srep11209 (PMC4462108; doi:10.1038/srep11209)

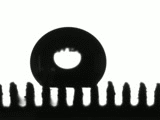

Supplement: Supplementary Information [file srep11209-s2.gif]
